# THE CHARM OF COUNTRY LIFE? IMPACT OF RURAL CHILDHOOD RESIDENCE ON PHYSICAL AND MENTAL HEALTH IN LATER LIFE

**DOI:** 10.1093/geroni/igad104.0076

**Published:** 2023-12-21

**Authors:** Danielle Nadorff, Angela Duck, Crystal Lim, Danielle Fastring

**Affiliations:** Mississippi State University, Mississippi State, Mississippi, United States; University of Mississippi Medical Center, Jackson, Mississippi, United States; University of Missouri, Columbia, Missouri, United States; William Carey University, Hattiesburg, Mississippi, United States

## Abstract

Background: Most studies of geographic health disparities are focused on adult rural residence. However, previous studies have shown that the residential area in which one grows up during childhood has lasting impacts on adult health. In one of the only studies to date to examine the impact of rural childhood residence on mental health in middle-aged and older adults, Murchland and colleagues (2019) evaluated inequalities by childhood residence and noted elevated depressive symptoms were more common among those living in rural areas compared to those living in non-rural areas. Aims: The current study expands the model proposed by Murchland and colleagues to include further antecedents related to rural childhood residence, and to include multiple outcomes of physical and mental health among middle-aged and older adults. Method: Participants included 4614 individuals aged 40 or older recruited as part of the Midlife in the United States (MIDUS) study. Results: Consistent with Murchland’s model, childhood rurality played an important part in middle-aged and older adults’ health, despite not having a direct influence. Rurality status was impacted by parental education level and SES during childhood and was associated with the level of education obtained by the participants (and thus their occupation), which played a direct role in their current health status. Mental and physical health had differential predictors. Limitations: The study was limited by its non-diverse sample and self-reported measures. Conclusion: Further research into the impact of childhood rurality on health is needed, utilizing comprehensive self-reported and observed outcome measures.

